# Asthma and Wheeze Prevalence among Nursing Professionals in Western Japan: A Cross-Sectional Study

**DOI:** 10.3390/ijerph121214997

**Published:** 2015-12-04

**Authors:** Jun Kurai, Masanari Watanabe, Hiroyuki Sano, Saeko Torai, Hirokazu Yanase, Tomoaki Funakoshi, Atsuko Fukada, Sachiko Hayakawa, Eiji Shimizu, Hiroya Kitano

**Affiliations:** 1 Department of Respiratory Medicine and Rheumatology, Faculty of Medicine, Tottori University, 36-1 Nishi-cho, Yonago 683-8504, Japan; junkurajun@gmail.com (J.K.); eiji@med.tottori-u.ac.jp (E.S.); 2 Department of Respiratory Medicine and Allergology, Faculty of Medicine, Kinki University, 377-2 Ohnohigashi, Osakasayama 589-0014, Japan; hsano@med.kindai.ac.jp; 3 Tottori Nursing Association, 318-1 Gotsu, Tottori 680-0901, Japan; torai@tottori-kangokyokai.or.jp; 4 Division of Nursing, Tottori University Hospital, 36-1 Nishi-cho, Yonago 683-8504, Japan; y-hiro0903@med.tottori-u.ac.jp (H.Y.); funakoshi@med.tottori-u.ac.jp (T.F.); atsuko-5@med.tottori-u.ac.jp (A.F.); 5 Department of Public Relations, Tottori University Hospital, 36-1 Nishi-cho, Yonago 683-8504, Japan; kangosk@med.tottori-u.ac.jp; 6 The Board of Directors, Tottori University, 4-101 Koyamachou-Minami, Tottori 68-8550, Japan; hkitano@med.tottori-u.ac.jp

**Keywords:** asthma, bed-making tasks, body mass index, ECRHS, employment period, latex allergy, nursing professionals, prevalence, smoking, wheeze

## Abstract

Although adult asthma is attributable to occupational factors, few reports are available on asthma prevalence among health care workers in Japan. The objective of this study was to estimate the prevalence of asthma and wheeze among Japanese nursing professionals. A cross-sectional study was conducted by postal survey using a translated version of the European Community Respiratory Health Survey questionnaire from April to June 2013. The analysis included 4634 nursing professionals (257 men and 4377 women) and the overall response rate was 84.8%. The prevalence of current asthma and wheeze were 10.7% (95% confidence interval (CI), 9.9%–11.7%) and 15.6% (95% CI, 14.5%–16.6%), respectively. More than one year of work experience as a nursing professional and more than one year of experience with bed-making tasks were associated with odds ratios (ORs) of 1.95 (95% CI, 1.12–3.39) and 1.64 (95% CI, 1.15–2.23) for wheeze, respectively. Current smoking was significantly associated with the presence of wheeze, with ORs of 2.27 for men (95% CI, 1.11–4.64) and 2.01 for women (95% CI, 1.54–2.64). Among female nurses, latex allergy was associated with wheeze (OR, 1.87; 95% CI, 1.56–2.23), as was body mass index ≥30 (OR, 2.76; 95% CI, 1.65–4.62). This study has provided the prevalence of asthma and wheeze among Japanese nursing professionals. Employment period, bed-making tasks, latex allergy, obesity, and smoking may be risk factors for prevalent wheeze among nursing professionals.

## 1. Introduction

Occupational exposures account for a substantial proportion of adverse respiratory health effects. Asthma has increased worldwide [1] and it has been recognized that certain occupational exposures are associated with some asthmas, which are referred to as work-related asthma (WRA) [2,3]. WRA includes occupational asthma (OA), which is caused by work, and work-exacerbated asthma (WEA) [2]. The overall prevalence of WRA has been difficult to establish with precision, partly because definitions, diagnostic criteria, and work settings can be ambiguous, and additionally because surveillance data are limited. Approximately 15% of cases of adult asthma are attributable to occupational factors [4].

Previous studies of occupational exposures among various groups of workers have suggested that asthma prevalence may be notably high among nursing professionals [5,6]. The large, multicenter European Community Respiratory Health Survey II (ECRHS II) study showed that nursing professionals had more than twice the risk of asthma (odds ratio = 2.22, 95% confidence interval (CI), 1.25 to 3.96), as compared with a reference group of professional, clerical, and administrative occupations [7]. Nursing professionals perform various forms of work, such as patient care activities that include drawing blood, mixing and administering medications, providing wound care and respiratory care, cleaning surgical and non-surgical instruments, mopping floors, assisting with invasive and other medical procedures, and assisting with anesthesia [8]. These activities potentially involve exposure to occupational hazards that pose risks of asthma. Several studies have indicated that the risk of asthma among nursing professionals is associated with chemical and cleaning-related tasks, as well as biological contaminants [5,6,8,9,10].

A recent nationwide cross-sectional study provided estimates of asthma prevalence and asthma symptom prevalence among Japanese adults [11]. However, in Japan, few studies have provided estimates of asthma risk among nursing professionals. To determine the prevalence of asthma among Japanese nursing professionals, a cross-sectional study was conducted in Western Japan using a standardized questionnaire. In addition to evaluating asthma and wheeze status, the questionnaire included assessments of different health care occupations, tasks, and employment periods.

## 2. Experimental Section

### 2.1. Study Design

The primary outcome variable was the prevalence of asthma and wheeze among nursing professionals. Using the European Community Respiratory Health Survey (ECRHS) questionnaire, a cross-sectional study was conducted in the neighboring Tottori and Shimane Prefectures, which are located in Southwest Japan. The population densities of Tottori and Shimane were 163.4/km^2^ and 103.9/km^2^, respectively. We asked the Nursing Association for assistance and received approval to submit the survey to the hospitals, clinics, and health care centers in Tottori and Shimane. Forty-five hospitals, 10 clinics, and 28 health care centers approved the survey. The nursing staffs at these hospitals, clinics, and health care homes were asked to participate in the study. If they provided written consent, a postal survey was performed. This study was conducted from October to December 2013. The study was approved by the institutional ethics committee of Tottori University (Ethics Committee of the Faculty of Medicine, Tottori University; Approval Number 2250).

### 2.2. Questionnaire

To evaluate asthma symptoms, we asked the same questions that had been included in the ECRHS. The Japanese version of the questionnaire was prepared based on the one-page ECRHS questionnaire, as well as page one of the two-page questionnaire that had been prepared for a stage one repeat study [11,12]. The validity of the questionnaire was established by translating the Japanese version back into English [13]. The questionnaire items can be found at http://www.ecrhs.org [14]. The questionnaire included additional questions on age, gender, height, weight, smoking history, presence of latex allergy, total duration of employment as a nursing professional, total duration of shift work, and duration of employment involving bed-making tasks in a medical setting. The presence of latex allergy was self-reported by the survey respondents. Total duration of employment as a nursing professional, total duration of shift work, and duration of employment involving bed-making tasks in a medical setting were categorized as (1) less than one year; (2) one year to less than five years; (3) five years to less than 10 years; (4) 10 years to less than 20 years; and (5) more than 20 years. 

A history of asthma as confirmed by a doctor was defined as an affirmative response to the question, “Have you ever had asthma?” (Q5), followed by an affirmative response to the question, “Was this confirmed by a doctor?” (Q5.1). Current asthma was defined as affirmative responses to each of the following questions: “Have you ever had asthma?” (Q5), followed by, “Was this confirmed by a doctor?” (Q5.1), and “Have you had at least one asthma-related symptom in the last 12 months?” [15]. If a respondent had been diagnosed with asthma by a physician and experienced positive asthma-related symptoms in the last 12 months, he or she was defined as currently having asthma [11]. Any subject who answered at least one of Q1–Q4 affirmatively was considered to have asthma-related symptoms. If a respondent had asthma-related symptoms in the past and remitted asthma-related symptoms, he or she was excluded from the current asthma category.

### 2.3. Statistical Analysis

Results are shown as means ± standard deviations (SDs). SPSS Statistical Software (Japanese version 21.0 for Windows; IBM Japan, Tokyo, Japan) was used for all statistical analyses. The prevalence of current asthma and the associated 95% CI were estimated for all participants aged 20–69 years. The Clopper–Pearson method was used to obtain the exact CI [16]. Fisher’s exact tests were performed to assess differences in prevalence between men and women. The differences in the prevalence of current asthma according to smoking history, presence of latex allergy, total duration of employment as a nursing professional, total duration of shift work, and duration of employment involving bed-making tasks in a medical setting were also evaluated. Fisher’s exact tests were also used to assess gender differences in prevalence within each age group. Univariate logistic regression analyses were used to calculate odds ratios and the associated 95% CIs for total duration of employment as a nursing professional, total duration of shift work, duration of employment involving bed-making tasks in a medical setting, latex allergy, smoking, and body mass index. All P-values are two-sided and the significance level was set to 0.05.

## 3. Results

A total number of 5463 nursing professionals provided written content and 4761 nursing professionals answered the questionnaire before the deadline for submission. Fifty-five out of 4761 subjects were excluded because of inadequate responses to Q1 of the questionnaire. Therefore, a total of 4634 subjects were included in the analysis, amounting to an overall response rate of 84.8%. Data on gender and age were not missing for any of these 4634 subjects. Table 1 presents the characteristics of the survey respondents, as grouped by gender, age, smoking history, presence of latex allergy, total duration of employment as a nursing professional, total duration of shift work, and duration of employment involving bed-making tasks in a medical setting. As expected, the study cohort was predominantly composed of women. For some study participants, data were missing on smoking history, presence of latex allergy, total duration of employment as a nursing professional, total duration of shift work, or duration of employment involving bed-making tasks in a medical setting; these participants were excluded from the analyses of associations with the prevalence of wheeze, total duration of employment, total duration of shift work, and duration of bed-making tasks in a medical setting. Similarly, these participants were excluded from the estimates of odds ratios for wheeze associated with total duration of employment as a nursing professional, total duration of shift work, duration of employment involving bed-making tasks in a medical setting, latex allergy, smoking, and body mass index.

**Table 1:** Descriptive characteristics of the study population.

Variables	Nursing Professionals (*n* = 4634)					
	Total		Men		Women	
	*n* (%)		*n* (%)		*n* (%)	
Gender			257	(5.5)	4377	(94.5)
Age (years)	40.4	±11.35	33.5	±7.97	40.8	±11.4
Smoking history						
Non-smoker	3743	(81.6)	107	(41.8)	3636	(83.9)
Past smoker	428	(9.3)	46	(18.0)	382	(8.8)
Current smoker	417	(9.1)	103	(40.2)	314	(7.2)
Presence of latex allergy	1026	(22.1)	33	(12.9)	990	(22.6)
Total employment as a nursing professional						
<1 year	184	(4.0)	24	(9.3)	160	(3.7)
1–4 years	628	(13.6)	72	(28.0)	556	(12.7)
5–9 years	750	(16.2)	69	(26.8)	681	(15.6)
10–19 years	1327	(28.6)	74	(28.8)	1253	(28.6)
≥20 years	1744	(37.6)	18	(7.0)	1726	(39.4)
Employment involving bed-making tasks in a medical setting						
<1 year	386	(8.3)	39	(15.2)	347	(7.9)
1–4 years	854	(18.5)	80	(31.1)	774	(17.7)
5–9 years	991	(21.4)	65	(25.3)	926	(21.2)
10–19 years	1301	(28.1)	58	(22.6)	1243	(28.5)
≥20 years	1094	(23.6)	15	(5.8)	1079	(24.7)
Body mass index						
<25	4031	(88.5)	200	(78.1)	3831	(89.1)
25.0–29.9	442	(9.7)	42	(16.4)	400	(9.3)
≥30	82	(1.8)	14	(5.5)	68	(1.6)
Total night shift work						
<1 year	375	(8.1)	39	(15.2)	336	(7.7)
1–4 years	890	(19.2)	80	(31.1)	810	(18.5)
5–9 years	980	(21.2)	67	(26.1)	913	(20.9)
10–19 years	1296	(28.0)	57	(22.2)	1239	(28.3)
≥20 years	1088	(23.5)	14	(5.4)	1074	(24.6)

Data are shown as mean ± standard deviation or number (%).

Table 2 shows the prevalence of asthma-related symptoms according to gender. The prevalences of current asthma and wheeze were 10.7% (95% CI, 9.9 to 11.7) and 15.6% (95% CI, 14.5 to 16.6), respectively (Table 3). There was no statistically significant difference between men and women in terms of current asthma or wheeze prevalence. Table 4 presents the prevalence of wheeze according to total duration of employment as a nursing professional. For both men and women, an employment period greater than one year was associated with a substantially elevated prevalence of wheeze. Duration of employment was significantly associated with wheeze prevalence in both men and women. Table 5 shows the prevalence of wheeze according to the duration of shift work. Shift patterns of work were significantly associated with the prevalence of wheeze in women, but not in men. Table 6 presents the prevalence of wheeze according to the duration of employment involving bed-making tasks in a medical setting. Duration of bed-making experience was significantly associated with the prevalence of wheeze in women.

Table 7 presents odds ratios for wheeze associated with total duration of employment as a nursing professional, total duration of shift work, and duration of employment involving bed-making tasks in a medical setting, latex allergy, smoking, and body mass index. For duration of employment as a nursing professional, shift work, and employment involving bed-making tasks in a medical setting, the reference categories of the odds ratios are <1 year of employment or experience. The reference category for the smoking variable analysis is never smokers, while the reference category for body mass index analysis is <25. The odds of prevalent wheeze were significantly elevated for female nursing professionals with more than one year of total employment as a nursing professional, shift work, and employment involving bed-making tasks in a medical setting. Similarly, obesity and latex allergy were significantly associated with the prevalence of asthma among female nursing professionals. As compared with those who never smoked, both male and female current smokers had significantly elevated odds of wheeze.

**Table 2:** Prevalence of asthma-related symptoms.

Variables	Total		Men		Women	
	% (95% CI)		% (95% CI)		% (95% CI)	
(Q1) Wheeze	15.6	(14.5–16.6)	17.1	(12.7–22.3)	15.5	(14.4–16.6)
(Q1.1) Wheeze with breathlessness	10.5	(9.6–11.4)	12.1	(8.3–16.7)	10.4	(9.5–11.3)
(Q1.2) Wheeze without a cold	9.5	(8.6–10.4)	12.1	(8.3–16.7)	9.3	(8.5–10.2)
(Q2) Waking with tightness in the chest	6.3	(5.6–7.0)	10.1	(6.7–14.5)	6.0	(5.4–6.8)
(Q3) Waking with an attack of shortness of breath	2.9	(2.4–3.4)	3.5	(1.6–6.5)	2.9	(2.4–3.4)
(Q4) Waking with cough	23.9	(22.7–25.2)	21.4	(16.5–26.9)	24.1	(22.8–25.4)
(Q5) Ever having asthma	17.4	(16.3–18.5)	21.0	(16.2–26.5)	17.2	(16.1–18.4)
(Q5.1) Ever having asthma confirmed by a doctor	16.9	(15.9–18)	19.5	(14.8–24.8)	16.8	(15.7–17.9)

CI: confidence interval. CIs were calculated with the Clopper-Pearson method.

**Table 3:** Prevalence of current asthma and wheeze.

Gender	Total		Men		Women	
	% (95% CI)		% (95% CI)		% (95% CI)	
Current asthma	10.7	(9.9–11.7)	10.5	(7.0–14.9)	10.8	(9.8–11.7)
Wheeze	15.6	(14.5–16.6)	17.1	(12.7–22.3)	15.5	(14.4–16.6)

CI: confidence interval. CIs were calculated with the Clopper-Pearson method.

**Table 4:** Prevalence of wheeze by duration of employment as a nursing professional.

Gender	Total Employment as a Nursing Professional										*p*-Value
	<1 Year % (95% CI)		1–4 Years % (95% CI)		5–9 Years % (95% CI)		10–19 Years % (95% CI)		≥20 Years % (95% CI)		
Men	4.2	(0.1–21.1)	27.8	(17.9–39.6)	17.4	(9.3–28.4)	13.5	(6.7–23.5)	5.6	(0.1–27.3)	0.027
Women	8.8	(4.9–14.2)	13.5	(10.8–16.6)	15	(12.4–17.9)	19.1	(17.0–21.4)	14.3	(12.7–16.1)	<0.001

CI: confidence interval. CIs were calculated with the Clopper-Pearson method.

**Table 5:** Prevalence of wheeze by duration of shift work.

Gender	Total Shift Work Employment										*p*-Value
	<1 Year % (95% CI)		1–4 Years % (95% CI)		5–9 Years % (95% CI)		10–19 Years % (95% CI)		≥20 Years % (95% CI)		
Men	10.3	(2.9–24.2)	22.5	(13.9–33.2)	19.4	(10.8–30.9)	14	(6.3–25.8)	7.1	(0.2–33.9)	0.338
Women	11.9	(8.6–15.9)	13.7	(11.4–16.3)	16.5	(14.2–19.1)	17.8	(15.7–20.0)	14.4	(12.4–16.7)	0.018

CI: confidence interval. CIs were calculated with the Clopper-Pearson method.

**Table 6:** Prevalence of wheeze by duration of employment involving bed-making tasks in a medical setting.

Gender	Employment Involving Bed-Making Tasks in a Medical Setting										*p*-Value
	<1 Year % (95% CI)		1–4 Years % (95% CI)		5–9 Years % (95% CI)		10–19 Years % (95% CI)		≥20 Years % (95% CI)		
Men	7.7	(1.6–20.9)	23.8	(14.9–34.6)	18.5	(9.9–30.0)	18.5	(9.9–30.0)	18.5	(9.9–30.0)	0.180
Women	10.4	(7.4–14.1)	14.2	(11.8–16.9)	15.3	(13.1–17.8)	15.3	(13.1–17.8)	15.3	(13.1–17.8)	0.001

CI: confidence interval. CIs were calculated with the Clopper-Pearson method.

**Table 7:** Logistic regression analysis of the prevalence of wheeze.

Variables	Odds Ratio (95% CI)			
	Men		Women	
Total duration of employment as a nursing professional				
≥1 year	5.21	(0.68–39.61)	1.95	(1.12–3.39)
Duration of shift work				
≥1 year	1.97	(0.66–5.84)	1.39	(0.99–1.95)
Duration of employment involving bed-making tasks in a medical setting				
≥1 year	2.78	(0.82–9.47)	1.64	(1.15–2.33)
Latex allergy	1.66	(0.70–3.98)	1.87	(1.56–2.23)
Smoking				
Past smoker	0.64	(0.20–2.06)	1.22	(0.92–1.62)
Current smoker	2.27	(1.11–4.64)	2.01	(1.54–2.64)
Body mass index				
≥25, <30	0.79	(0.31–2.01)	1.30	(0.99–1.70)
≥30	1.29	(0.34–4.85)	2.76	(1.65–4.62)

CI: confidence interval; Odds ratios for the prevalence of wheeze were estimated. For durations of employment as a nursing professional, shift work, and employment involving bed-making tasks in a medical setting, the reference categories of the odds ratios are <1 year of employment or experience. The reference category for the smoking variable are those who never smoked. The reference category for body mass index is <25.

## 4. Discussion

OA has become one of the most common forms of occupational lung disease in many industrialized countries [17,18]. A high rate of asthma has been observed in nursing professions, as compared with professional, clerical, and administrative occupations [7]. However, few reports are available on the prevalence of asthma among nursing professionals in Japan. This study estimated the prevalence of asthma and wheeze among Japanese nursing professionals using the ECRHS questionnaire. Our key findings were that the durations of employment as a nursing professional, shift work, employment involving bed-making tasks were significantly associated with the presence of wheeze. These results provide fundamental information regarding OA in Japanese nursing professionals.

Relatively few studies have used standardized questionnaires to investigate the prevalence of asthma and asthma symptoms in Asian countries. Recently, Fukutomi *et al.* used the ECRHS questionnaire to conduct a population-based cross-sectional study of the prevalence of asthma and wheeze in Japanese adults [11]. They found that the prevalence of asthma and wheeze among Japanese adults aged 27–79 years were 4.2% (95% CI, 9.7% to 10.5%) and 10.1% (95% CI, 9.7% to 10.5%), respectively. In the current study, we found that asthma and wheeze prevalence were higher among Japanese nursing professionals than was reported in this previous study of the adult population of Japan. Several studies have demonstrated that nursing professionals in Western countries also have a high prevalence of asthma and wheeze [5,6,7,8,9,10]. The results of the current study suggest that occupational exposures during nursing work may account for a substantial proportion of asthma cases among Japanese nursing professionals.

More than 300 natural and synthetic chemicals have been reported to cause OA (with latency) [3]. Regarding OA, it is suspected that the onset of asthma symptoms related to a sensitizer usually occurs between weeks and years after a latent period of exposure to a causal agent [2]. The current study found a significant association between the total duration of employment as a nursing professional and the prevalence of wheeze. Additionally, when the duration of employment exceeded one year, the prevalence of wheeze was substantially elevated. These results suggest that causal agents of asthma are likely to be present in the nursing work environment in Japan. Within a relatively short time since they had begun nursing work, nursing professionals in Japan may have an elevated risk of OA. In some cases, the occurrence of asthma and wheeze in early stages of employment may lead to a departure from nursing work. For longer employment durations, the prevalence of current asthma and wheeze may, therefore, be underestimated.

Mattresses can be a source of a range of chemicals, including volatile organic compounds, plasticizers, flame retardants, and unreacted isocyanates [19,20]. In addition, bedding, mattresses, and pillows harbor a wide variety of particles, many of which are biological. Dust found in mattresses contains a multitude of organisms and their associated allergens. Mattress dust also hosts house dust mite allergens [21,22,23]. Several studies have demonstrated relationships between levels of house dust mite allergen in mattresses and allergic respiratory symptoms, such as the onset of wheezing [24,25].

Although stress is a related factor in many explanations of the origins of asthma, the role of stress in the development and exacerbation of asthma remains poorly understood. Several studies have reported that stress has negative effects on the immune system, which may trigger asthma [26,27]. Sleep has important homeostatic functions, and circadian rhythms organize physiology and behavior on a daily basis to ensure optimal function. Sleep deprivation and circadian disruption can be stressors and can additionally enhance other stressors that have consequences for the brain and many systems of the body. Previous studies have demonstrated the effects of work-related stress on sleep problems [28,29,30]. Day and night shift schedules may be associated with the development of asthma, but few studies have investigated the relationship between shift work and asthma. Therefore, we studied the association between the total duration of shift work and the prevalence of wheeze, finding that the duration of shift work was significantly associated with the presence of wheeze. Further studies are needed to investigate the effects of shift work on the development of asthma in greater detail.

Various risk factors for OA have been established [3]. Atopy is a predisposing factor in workers exposed to high-molecular-weight agents, but it is a weak predictor of sensitization and the development of OA [31]. Importantly, several studies have found that natural rubber latex has become the most common cause of OA in industrialized countries [5,32,33,34]. In the current study, we also found a significant association between latex allergy and wheeze. Larese *et al.* reported that a changeover from powdered to non-powdered latex gloves can reduce latex-related symptoms. It may also be possible to reduce development of OA by using non-latex gloves [35].

Cleaning products and disinfectants are commonly used in both occupational and non-occupational settings. In medical settings, the many chemical products that are involved include topical cleansers and antiseptics that are applied to the patient’s skin, glutaraldehyde for cold sterilization of medical instruments, and all-purpose general cleaners, such as bleach [8]. Rosenman *et al.* reported that 12% of work-related asthma cases were related to the use of cleaning products [36]. Arif *et al.* have also shown that, in comparison with other healthcare professionals, nursing professionals have an increased risk of developing new-onset asthma due to the chemicals and compounds that are used during patient care activities, medical instrument cleaning/disinfection, and general cleaning [8]. Additionally, cigarette smoking has been reported to be associated with the development of OA in workers who are exposed to anhydride compounds [37]. In the present study, we found a significant relationship between the prevalence of wheeze and cigarette smoking. The development of OA may involve interaction effects between cigarette smoking and exposure to anhydride compounds. Understandably, cigarette smoking can increase the risk of sensitization to agents causing OA [38].

The present study evaluated the risks of wheeze that were associated with body mass index, smoking history, presence of latex allergy, total duration of employment as a nursing professional, total duration of shift work, and duration of employment involving bed-making tasks in a medical setting. The risks of wheeze differed significantly between men and women. It is possible that these differences resulted from the different numbers of men and women who were included in the study. On the other hand, a review of more than 60,000 subjects with asthma in a large healthcare organization showed that utilization of inhaled corticosteroids, routine hospital visits, emergency visits, and hospitalization appeared to be more common in women aged 23–64 years than in men [39]. Additionally, women in some professions have greater risks of OA than do men [40]. Female sex may be associated with increased OA risk. The mechanisms underlying the striking sex difference merit further investigation.

The ECRHS questionnaire does not include a clear definition of current asthma. Previous studies using the ECRHS questionnaire employed various definitions of current asthma. Therefore, in this study, we estimated the associations between the prevalence of wheeze and body mass index, smoking history, presence of latex allergy, total duration of employment as a nursing professional, total duration of shift work, and duration of employment involving bed-making tasks in a medical setting. Our study was limited by its reliance on a questionnaire of symptoms to diagnose asthma and asthma-related symptoms. This approach may lead to an overestimation of asthma prevalence. Similarly, the diagnosis of latex allergy depended on self-reporting by respondents. Further investigation is needed, including skin-prick tests. One further limitation to this study is that we were unable to survey the number of employees in each hospital or clinic. Therefore, we were unable to investigate the effects on the prevalence of current levels of asthma and wheeze that resulted from differences in employment between hospitals, clinics, and health care centers.

## 5. Conclusions

In conclusion, this cross-sectional study used a Japanese version of the ECRHS questionnaire to determine that the prevalence of current asthma and wheeze among Japanese nursing professionals, which were 10.7% and 15.6%, respectively. As compared with Japanese adults, Japanese nursing professionals have more than 1.5 times the risk of prevalent asthma. Further, durations of employment as a nursing professional, shift work, and employment involving bed-making tasks were each associated with the presence of wheeze.
